# A probabilistic deep learning approach for choroid plexus segmentation in autism spectrum disorder

**DOI:** 10.1038/s44277-026-00056-1

**Published:** 2026-01-30

**Authors:** Filippo Bargagna, Thomas M. Morin, Ya-Chin Chen, Ylind Lila, Chieh-En J. Tseng, Maria F. Santarelli, Nicola Vanello, Christopher J. McDougle, Jacob M. Hooker, Nicole R. Zürcher

**Affiliations:** 1 A.A. Martinos Center for Biomedical Imaging, Department of Radiology, Massachusetts General Hospital, Harvard Medical School, Boston, MA USA; 2 Dipartimento di Ingegneria dell’Informazione, University of Pisa, Pisa, Italy; 3 Bioengineering Unit, Fondazione Toscana Gabriele Monasterio, Pisa, Italy; 4 Department of Psychology, Brandeis University, Waltham, MA USA; 5 Lurie Center for Autism, Massachusetts General Hospital, Lexington, MA USA; 6 Department of Radiology and Nuclear Medicine, University Hospital Basel, Basel, Switzerland; 7 CNR Institute of Clinical Physiology, Pisa, Italy

**Keywords:** Predictive markers, Neuroscience

## Abstract

The choroid plexus serves as the primary barrier between the brain’s blood and cerebrospinal fluid and mediates neuroimmune function. A subset of individuals with autism spectrum disorder (ASD) may exhibit morphological alterations of the choroid plexus. However, to power larger population analyses, an automated tool capable of accurately segmenting the choroid plexus based on magnetic resonance imaging (MRI) is needed. Automated Segmentation of CHOroid PLEXus (ASCHOPLEX) is a deep learning tool that enables finetuning using new, patient-specific, training data, allowing its usage across cohorts for which the model was not originally trained. We evaluated ASCHOPLEX’s generalizability to individuals with ASD by performing finetuning on a local dataset of ASD and control (CON) participants. To assess generalizability, we implemented a probabilistic version of the algorithm, which allowed us to quantify the uncertainty in choroid plexus segmentation and evaluate the model’s confidence. ASCHOPLEX generalized well to our local dataset, in which all participants were adults. To further assess its performance, we tested the algorithm on the Autism Brain Imaging Data Exchange (ABIDE) dataset, which includes data from children and adults. While ASCHOPLEX performed well in adults, its accuracy declined in children, suggesting limited generalizability to different age groups without additional finetuning. Our findings show that the incorporation of a probabilistic approach during finetuning can strengthen the use of this deep learning tool by providing confidence metrics which allow assessing model reliability. Overall, our findings demonstrate that ASCHOPLEX can generate accurate choroid plexus segmentations in previously unseen data.

## Lay Summary

The choroid plexus plays an important role in brain health and immunity and may be altered in autism spectrum disorder (ASD). To analyze large imaging datasets, a method to automatically delineate this structure is needed. We adapted an existing artificial intelligence tool, ASCHOPLEX, for use in individuals with ASD and made it probabilistic to probe the confidence of its automated segmentations. The results show that ASCHOPLEX can generate accurate choroid plexus segmentations in ASD.

## Introduction

The choroid plexus forms the brain’s blood-cerebrospinal fluid (CSF) barrier (BCSFB) and is responsible for producing CSF. Importantly, the choroid plexus is involved in neuroimmune signaling, triggering immune responses in the central nervous system [1–4]. Alterations to choroid plexus morphology are thought to reflect neuroinflammation [5], and have been found across a range of neurological and psychiatric conditions [6–18]. Previous work has indicated that morphological differences in the choroid plexus may also be apparent in autism spectrum disorder (ASD) [19–21]. These results complement hypotheses that abnormal neuroinflammatory responses may be a driver of pathology in a subset of individuals with ASD [22]. Additional work in larger cohorts is needed to understand the extent to which molecular and morphological changes to the choroid plexus, such as signs of neuroinflammation, are present in autistic individuals.

Currently, the gold standard for segmenting the choroid plexus *in vivo* is to manually trace the region on a T1-weighted (T1w) or Fluid Attenuated Inversion Recovery (FLAIR) magnetic resonance (MR) image. Manually tracing the choroid plexus on these images is (1) difficult, due to low contrast between ventricular CSF and the choroid plexus particularly on T1w images, (2) laborious, requiring highly trained individuals several hours to trace a single subject, and (3) subjective, due to highly variable choroid plexus anatomy present across individuals. Given there is no standardized, scalable protocol for manual segmentation, automated methods are needed to study the choroid plexus in larger cohorts.

Several automated segmentation methods have been developed for efficiently generating choroid plexus labels on larger MR datasets, reporting near-human-levels of accuracy when compared to labels generated manually by radiologists [13, 16–18, 23–25]. While these approaches demonstrate incredible progress, it is difficult to assess how well they generalize to new populations (e.g. different age groups, other psychiatric/neurological conditions, etc.). For example, of the seven tools cited above, FreeSurfer is the only method that has been used in ASD [19, 20, 24]. While FreeSurfer excels at segmenting cortical and subcortical brain regions, it has not been specifically trained to segment the choroid plexus, and new automated methods show major improvements in choroid plexus segmentation [13, 16–18, 23, 25]. Assessing the performance, generalizability, and uncertainty of a novel automated segmentation procedure that leverages deep learning has not been done in ASD.

The Automated Segmentation of CHOroid PLEXus (ASCHOPLEX) is a recently published method for choroid plexus segmentation that showed a high Dice score when compared to manual segmentation [25]. ASCHOPLEX uses an ensemble of deep learning models trained on healthy adult controls and adults with relapsing-remitting multiple sclerosis (see methods for details) [25]. To facilitate the generalization of ASCHOPLEX to new cohorts, an implemented finetuning procedure allows the user to supply a small amount of additional training data to the model. Visani and colleagues demonstrated that ASCHOPLEX successfully generalized to a new dataset consisting of adults with and without depression [25].

Assessing the generalization of machine learning algorithms typically requires the creation of manually-labeled “ground truth” datasets – a tedious process that quickly becomes intractable in larger datasets. More recently, probabilistic machine learning approaches have been employed to quantify model uncertainty, allowing assessment of a model’s generalizability to new datasets where “ground truth” labels are not available (reviewed in [26]). Here we tested the extent to which ASCHOPLEX could be generalized to groups with ASD. First, following a finetuning procedure, we tested the generalization of ASCHOPLEX in a local cohort of adult ASD and control (CON) participants (n = 65, ages 18 – 40 years), for whom manual segmentations of the choroid plexus had previously been generated [21]. Then, we tested whether ASCHOPLEX, finetuned on our local cohort of ASD and CON participants, can generalize to a much larger cohort of adults and children with and without ASD from the Autism Brain Imaging Data Exchange (ABIDE) dataset (n = 2226, ages 5 – 65 years). To evaluate generalization performance, we implemented a probabilistic version of ASCHOPLEX and assessed the algorithm’s level of uncertainty across both the local and the ABIDE datasets. Our primary hypothesis was that ASCHOPLEX would exhibit the least uncertainty in individuals most similar to the training and finetuning datasets. Specifically, we hypothesized that ASCHOPLEX would show less uncertainty for ASD and CON adults compared to ASD and CON children. Overall, this study investigated whether ASCHOPLEX can generalize choroid plexus segmentation to ASD participants with finetuning, while also exploring the advantage of uncertainty quantification to assess its reliability across datasets.

## Methods

### Local dataset

For finetuning and model evaluation we used T1-weighted multi-echo magnetization prepared rapid gradient echo (MEMPRAGE) high-resolution anatomical magnetic resonance imaging (MRI) acquisitions (voxel size = 1.0 × 1.0 × 1.0 mm, TR = 2530 ms, TE[1–4] = 1.66 ms, 3.53 ms, 5.3 ms, 7.27 ms, matrix = 280 × 280 × 208, sagittal plane, 208 slices, flip angle = 7.0 deg) from a local dataset comprised of 65 participants: 36 ASD (24 males, 12 females) and 29 CON (19 males, 10 females) [21, 27–29]. All participants were 18 to 40 years old and had an intelligence quotient (IQ) at or above moderate intellectual disability (IQ ≥ 35) for autistic individuals, and at or above normal IQ (IQ ≥ 85) for CON. MRI scans were performed on a 3 Tesla TIM Trio MRI scanner at the Athinoula A. Martinos Center for Biomedical Imaging. ASD diagnosis was based on the Diagnostic and Statistical Manual of Mental Disorders 5th edition [30] and assessed by board certified psychiatrists and corroborated by Autism Diagnostic Interview-Revised (ADI-R) and Autism Diagnostic Observation Schedule-2 (ADOS-2), module 4, as reported previously [21, 27–29]. The Massachusetts General Brigham institutional review board (IRB) approved the protocol for the study and all participants provided informed consent. Informed consent was provided by participants or their legally authorized representative (LAR; as appropriate for individuals with ASD and impaired capacity to provide consent). Participants with ASD provided written assent when LAR consent was obtained.

Manual segmentations of the choroid plexus in the lateral ventricles have been previously described [21]. The choroid plexus was traced in OsiriX (https://www.osirix-viewer.com) by a diagnostic radiology trainee (YL) using each participant’s T1-weighted MEMPRAGE scan. A neuroimaging researcher (CJT, > 10 years in MRI research) conducted a second visual quality control [21]. Manual segmentations were performed by adjusting the contrast of the image to adequately visualize the choroid plexus, tracing the morphology of the choroid plexus of the lateral ventricles slice by slice in the axial plane, and adjusting the boundaries on the coronal and sagittal planes to ensure that the segmentation did not include CSF or brain tissue. Representative examples of manual choroid plexus segmentations from the local dataset are presented in Figure S3.

### ABIDE dataset

The Autism Brain Imaging Data Exchange (ABIDE) dataset is a publicly available collection of brain imaging data from 2226 subjects, including 1060 subjects with ASD and 1166 CON participants [31, 32]. The data was collected from 24 international brain imaging laboratories with a range of scanner types. ABIDE I comprises acquisitions from 17 sites, for a total of 1112 subjects (539 individuals with ASD and 573 CON; ages 7–64 years) [31]. ABIDE II comprises 1114 additional subjects from 19 sites (521 individuals with ASD and 593 CON; ages 5–64 years) [32]. We applied a manual quality control (QC) procedure in which two independent raters (TMM, YC) performed a visual inspection and judged the quality of each subject’s T1-weighted image. Raters completed an initial pass-through, viewing each structural image and flagging any subject with missing data, major scanner noise/artifact, partial field of view (FOV), incorrect image orientation, extreme motion artifact, or other visible artifact (e.g. major signal dropout). After the first pass-through, the raters re-examined any subject on which there was disagreement and came to a final decision regarding inclusion/exclusion. Following this procedure, 410 subjects were excluded from the original ABIDE sample (18.53% of the total). A CSV file listing the included/excluded ABIDE subjects is available in the Supplementary Materials. The scans of 1802 subjects passed the QC procedure. The final sample consisted of 708 males with ASD, 109 females with ASD, 774 male CON, and 252 female CON.

### Automatic segmentation of CHOroid PLEXus (ASCHOPLEX)

ASCHOPLEX is a model ensemble developed by Visani and colleagues [25]. Detailed information about the ASCHOPLEX model and its finetuning procedure is presented in the Supplementary Materials. To apply the model in the context of ASD, we performed finetuning on a representative subsample of our local dataset. The sample used for finetuning consisted of twelve subjects: six subjects for training (n = 3 ASD [2 males, 1 female], n = 3 CON [2 males, 1 female]), and six subjects for validation (n = 3 ASD [2 males, 1 female], n = 3 CON [2 males, 1 female]). After finetuning, we tested the model’s performance on the remaining 53 subjects using the Dice similarity coefficient to quantify the model’s accuracy. The 53 subjects in the test set consist of 30 ASD (20 males, 10 females) and 23 CON (15 males, 8 females).

### A probabilistic implementation of ASCHOPLEX

Because ASCHOPLEX is an ensemble of deterministic neural networks [25], information regarding model uncertainty is limited to the differences observed between the five ensemble models. To assess model generalizability, a more fine-grained metric is required. To measure uncertainty, we implemented a probabilistic version of the ASCHOPLEX model, which provides information about the model’s confidence in each inference. To make ASCHOPLEX probabilistic, we opted for a Monte Carlo Dropout (MC Dropout) approach. In essence, we enabled dropout layers prior to finetuning (as having dropout active during finetuning may help prevent potential mode-collapse, overfitting and catastrophic forgetting) and during inference in each of the five models that make up the ensemble [33]. Enabling dropout refers to randomly removing units from a neural network with a certain probability. We selected a dropout probability of 0.1. All dropout layers, where originally present within the architectures, were activated. Dropout rates of 0.1, 0.25, 0.4, and 0.5 were empirically tested on the local dataset. The dropout rate of 0.1 was selected for having the highest Dice coefficient while still allowing for enough variability to gather significantly detailed uncertainty metrics (see Figure S1). Neural networks with dropout layers can be considered approximate Bayesian Neural Networks (BNN), making them probabilistic [34]. In addition to enabling dropout in the probabilistic approach, we also modified the ASCHOPLEX post-processing code to output a probability for a given voxel to be labeled choroid plexus (range 0 – 1), rather than only creating binary labels through direct thresholding at 0.5 (0: background, 1: choroid plexus) as done in the original implementation, where voxel-wise probabilities are not preserved.

### Assessing model performance using the local dataset

**Fig. 1:**
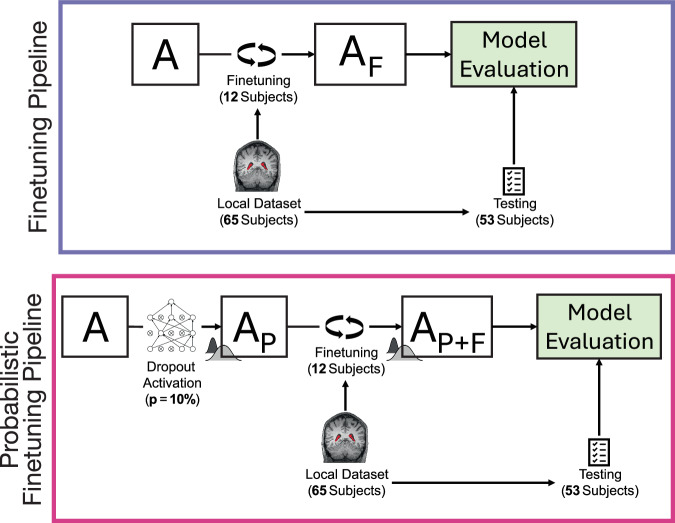
Pipeline for the finetuning of ASCHOPLEX for the deterministic (top) and probabilistic (bottom) variants. The probabilistic model is obtained by activating the dropout layers within the model ensemble prior to finetuning. Subsequently, the pipeline is identical for both models: a local dataset (n = 65) is split into two groups, with one group of 12 subjects used for finetuning and one group of 53 subjects used for model evaluation.

**Fig. 2:**
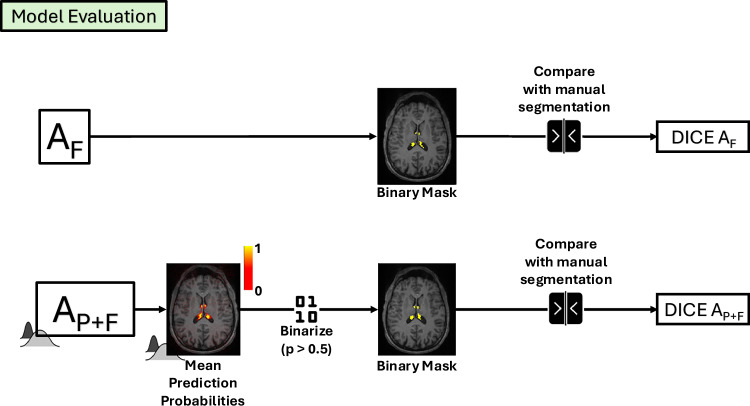
Pipeline for the model evaluation of the Finetuned (top) and Probabilistic Finetuned (bottom) version of the ASCHOPLEX algorithm. To obtain the binary segmentation masks from the probabilistic model we thresholded the output mean prediction probabilities at 0.5 (50%). Subsequently, the pipeline was identical for both models: the binary masks were compared with the available manual segmentations to compute the Dice coefficient.

We assessed the performance of two versions of ASCHOPLEX, see Fig. 1: (1) the default deterministic version and (2) our adapted probabilistic version. Choroid plexus labels were generated from both pipelines, and the models were evaluated using 53 held-out subjects from the local dataset. The model evaluation procedure is shown in Fig. 2. Because the probabilistic pipeline yields a different choroid plexus label each time it is run, we repeated the inference on the test set twenty times (for each of the five models in the ASCHOPLEX ensemble), resulting in 100 probabilistic choroid plexus masks for each subject. The final segmentation from the probabilistic pipeline was obtained by generating the mean image from the 100 probabilistic labels and binarizing with a threshold of 0.5. The threshold of 0.5 was selected based on its common use for binarizing two-category decisions. A follow-up analysis showed that alternative thresholds above 0.2 did not result in statistically different Dice coefficients across the held-out sample, see Figure S2. In the deterministic pipeline, a binary mask is produced through majority voting of the five models in the ensemble. The binary masks were then compared to manually segmented labels for each subject (considered ground-truth) and Dice similarity coefficients were generated. For comparison with an automated segmentation using a standard automated segmentation approach that does not rely on deep learning, we generated segmentations using FreeSurfer v.6.0.0.

### Assessing model uncertainty on local and ABIDE dataset

For the local dataset we generated 100 stochastic segmentations per subject (20 Monte-Carlo (MC) dropout passes × 5 trained ensemble models). For the ABIDE dataset, we applied the same full sampling scheme (20 × 5 = 100 samples) to a representative subset of 86 subjects, and a lighter scheme (5 × 5 = 25 samples) to the full ABIDE cohort for computational feasibility. For each subject, voxel-wise predictive mean probabilities were computed as the average across all stochastic samples. We then estimated four uncertainty metrics at each voxel:

1. Total predictive entropy (combined uncertainty):
  $${H}_{{total}}\left(v\right)=-\bar{p}\left(v\right) \, {\cdot } \, {ln} \, \bar{p}\left(v\right)-[1-\bar{p}\left(v\right)] \, {\cdot } \, {ln}[1-\bar{p}\left(v\right)]$$
2. Expected per-sample entropy (aleatoric uncertainty proxy):
  $${E}_{s}\left[{H}_{s}(v)\right]=\left(1/S\right){\sum }_{s}\big[-{p}_{s}(v) \, {\cdot } \, {ln} \, {p}_{s}(v)-(1-{p}_{s}(v)) \, {\cdot } \, {ln}(1-{p}_{s}(v))\big]$$
3. Mutual information (epistemic uncertainty):
  $${MI}\left(v\right)={H}_{{total}}(v)-{E}_{s}\left[{H}_{s}(v)\right]$$
4. Standard deviation of predictions (non-separated dispersion):
  $$\sigma \left(v\right)=\sqrt{\left[(1/S){\sum }_{s}{\left({p}_{s}\left(v\right)-\bar{p}\left(v\right)\right)}^{2}\right]}$$

where $\bar{p}\left(v\right)$ is the mean predicted probability at voxel $v$ over all stochastic samples, and ${p}_{s}\left(v\right)$ is the probability from sample $s$. This information-theoretic decomposition corresponds to the Bayesian Active Learning by Disagreement (BALD) criterion, separating total uncertainty into aleatoric and epistemic components [34–36], while the standard deviation map provides an intuitive dispersion index without theoretical separation. In this context, the aleatoric term corresponds to the expected conditional entropy of the posterior predictive distribution and captures uncertainty that is irreducible under the learned model. This should be distinguished from approaches that explicitly parameterize data-dependent noise at the likelihood level; here, aleatoric uncertainty is inferred at the level of the posterior predictive rather than directly modeled. Enhancing an ensemble by enabling MC dropout for each model allows us to gain insight on both inter-model and intra-model variability. The effect of architecture and loss landscape of each model is united with single-model variability in inference from run-to-run. This gives an extra dimension on which to compute uncertainty metrics and allows for an increase in the number of samples that can be collected. Each voxel prediction ${\bar{p}}_{m,k}\left(v\right)$ comes from model m and dropout realization k.

For each subject and each metric, voxel-wise uncertainty maps were then masked by the binarized choroid plexus label (described above). The final uncertainty metric for each subject was defined as:

$$C=\frac{{\sum }_{0}^{n}({U}\left(p_{i}\right){* b}_{i})}{{\sum }_{0}^{n}{b}_{i}}$$

where $U\left({p}_{i}\right)$ is the uncertainty metric computed on the 25 or 100 probabilities for voxel $i\epsilon \left[1,{n}\right|$, and ${b}_{i}$ is a binary value indicating whether a voxel belongs to choroid plexus (1) or not (0). Because our local dataset contained only adults aged 18–40 years, we assessed model generalizability to the ABIDE cohort by comparing the uncertainty measures across children (ages 5 to 17, 11.79 + /- 2.81) and adult participants (ages 18 to 64, 27.17 + /- 9.38) in ABIDE (see Fig. 4).

### Statistical analysis

To assess model performance, Dice coefficients were compared across segmentation methods (FreeSurfer, ASCHOPLEX deterministic without finetuning, ASCHOPLEX deterministic with finetuning, ASCHOPLEX probabilistic with finetuning) compared with manual segmentation of the choroid plexus using the local dataset. Dice coefficient is defined as:

$${Dice}=\frac{2 {\sum }_{0}^{n}{a}_{i}{m}_{i}}{{\sum }_{0}^{n}{a}_{i}+{\sum }_{0}^{n}{m}_{i}}$$

where ${a}_{i}=1$ if a voxel $i$ is labeled as choroid plexus on the automated segmentation, and ${a}_{i}=0$ if not; and ${m}_{i}=1$ if a voxel $i$ is labeled as choroid plexus on the manual segmentation and ${m}_{i}=0$ if not; for images that contain $n$ voxels total.

Additional metrics used to assess model performance include Hausdorff distance (as defined in [37]), volume similarity, and Pearson correlation of volumes between manual and automated methods. These metrics are explained in detail and presented in the Supplementary Materials (see Figure S5).

A linear mixed effects model was used to assess whether a given segmentation procedure exhibited a performance bias for either diagnostic group (ASD vs. CON) or sex (male vs. female). In the model, Dice coefficient was the dependent variable, with fixed effects for segmentation procedure, sex, diagnosis, and the interactions between segmentation procedure, sex, and diagnosis and a random effect of intercept [Dice ~ segmentation * sex * diagnosis + (1 | subject)]. The linear mixed effects model was implemented in R (v. 4.2.2) using the lme4 package. Post-hoc independent-samples t-tests were then used to compare the Dice coefficients for diagnostic group and sex. All p-values were corrected for multiple comparisons using FDR-correction.

## Results

### Finetuning improves segmentation accuracy in autistic participants

**Fig. 3:**
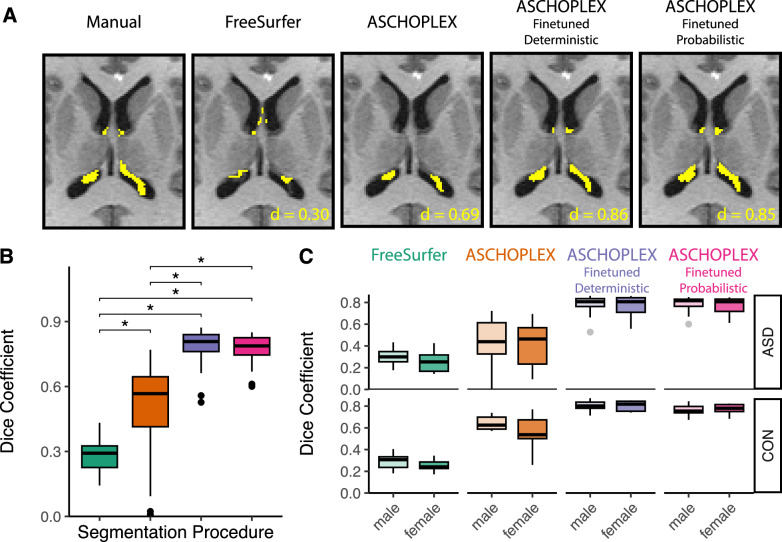
Example of the choroid plexus segmentation for an individual subject from the local dataset for each of the methods. The subject shown is the subject with the best Dice coefficient for ASCHOPLEX Finetuned Probabilistic (**A**). Dice coefficients for the automated segmentation methods vs. manual segmentation in the 53 held-out participants from the local dataset (**B**). Dice coefficients compared across sex and diagnosis for each of the segmentation methods for participants from the local dataset (**C**).

Example segmentations from each method are shown for a single participant in Fig. 3a (and Figure S4 for failure-mode characterization). The linear mixed effects model demonstrated a significant effect of segmentation procedure on Dice coefficient where all segmentations performed better than FreeSurfer (Non-Finetuned ASCHOPLEX: b = 0.15, t = 4.04, p < 0.001; Deterministic Finetuned ASCHOPLEX: b = 0.52, t = 13.02, p < 0.001; Probabilistic Finetuned ASCHOPLEX: b = 0.52, t = 13.06, p < 0.001). Post-hoc pairwise T-tests demonstrated that the non-finetuned ASCHOPLEX model exhibited better performance than FreeSurfer’s segmentation (T(53) = 7.89, p < 0.001). Both the deterministic and probabilistic finetuned ASCHOPLEX models showed significantly better performance than both FreeSurfer (Deterministic vs. FreeSurfer: T(53) = 39.0, p < 0.001; Probabilistic vs. FreeSurfer: T(53) = 40.6, p < 0.001) and the non-finetuned model (Deterministic vs. No Finetuning: T(53) = 9.93, p < 0.001; Probabilistic vs. No Finetuning: T(53) = 9.54, p < 0.001). Furthermore, analyses confirmed that using the probabilistic model instead of the deterministic model did not result in any significant reduction in model performance (T(53) = 1.16, p = 0.25). (see Fig. 3b).

### Model performance bias assessment

The linear mixed effects model also tested for potential performance biases for diagnosis (ASD vs. CON) and/or sex (male vs. female). The model demonstrated a significant interaction effect between diagnosis and segmentation procedure such that non-finetuned ASCHOPLEX had a worse Dice coefficient for ASD compared to CON participants (b = 0.15, t = 2.70, p < 0.01). The model did not detect a significant effect of or interaction with sex on Dice coefficient. Posthoc T-tests demonstrated that FreeSurfer did not show any significant bias, with comparable Dice coefficients across diagnostic groups and sexes (ASD vs. CON: T(30,23) = 0.40, p = 0.69; ASD Male vs. ASD Female: T(20,10) = −1.48, p = 0.16; CON Male vs. CON Female: T(15,8) = −1.71, p = 0.10). Additionally, without finetuning, ASCHOPLEX showed significantly better performance on CON participants compared to ASD participants (T(23,30) = 4.04, p < 0.001). However, the non-finetuned ASCHOPLEX did not show any significant bias for sex (ASD Male vs. ASD Female: T(20,10) = −0.281, p = 0.78; CON Male vs. CON Female: T(15, 8) = −1.59, p = 0.14). The deterministic finetuned ASCHOPLEX did not show a significant bias for diagnosis or sex (ASD vs. CON: T(30, 23) = 1.39, p = 0.17, ASD Male vs. ASD Female: T(20,10) = −0.52, p = 0.61; CON Male vs. CON Female: T(15,8) = −0.092, p = 0.93). Similarly, the probabilistic finetuned ASCHOPLEX did not show a significant bias for diagnosis or sex (ASD vs. CON: T(30,23) = 0.718, p = 0.48; ASD Male vs. ASD Female: T(20,10 = −0.59, p = 0.56); CON Male vs. CON Female: T(15, 8) = 0.395, p = 0.70). See Table 1 and Fig. 3c.

**Table 1:** Model Performance Bias Assessment (Dice Coefficient).

Model	Group	Mean [IQR]	T-Test
FreeSurfer	ASD Female	0.26, [0.17, 0.32]	T(11, 23) = −1.48, p = 0.42
	ASD Male	0.3, [0.25, 0.35]	
	CON Female	0.25, [0.23, 0.29]	T(9, 18) = −1.71, p = 0.42
	CON Male	0.29, [0.24, 0.33]	
	All ASD	0.29, [0.22, 0.34]	T(35, 28) = 0.4, p = 0.69
	All CON	0.28, [0.23, 0.32]	
ASCHOPLEX	ASD Female	0.41, [0.23, 0.57]	T(9, 19) = −0.28, p = 0.89
	ASD Male	0.43, [0.33, 0.61]	
	CON Female	0.56, [0.5, 0.67]	T(7, 14) = −1.59, p = 0.42
	CON Male	0.64, [0.59, 0.7]	
	All ASD	0.42, [0.31, 0.59]	T(29, 22) = −4.04, p < 0.05
	All CON	0.61, [0.57, 0.7]	
ASCHOPLEX Finetuned, Deterministic	ASD Female	0.77, [0.71, 0.84]	T(9, 19) = −0.52, p = 0.89
	ASD Male	0.79, [0.76, 0.84]	
	CON Female	0.8, [0.75, 0.84]	T(7, 14) = −0.09, p = 0.93
	CON Male	0.8, [0.78, 0.84]	
	All ASD	0.78, [0.75, 0.84]	T(29, 22) = −1.39, p = 0.34
	All CON	0.8, [0.77, 0.84]	
ASCHOPLEX Finetuned, Probabilistic	ASD Female	0.77, [0.72, 0.83]	T(9, 19) = −0.59, p = 0.89
	ASD Male	0.79, [0.76, 0.83]	
	CON Female	0.77, [0.75, 0.81]	T(7, 14) = 0.4, p = 0.89
	CON Male	0.77, [0.74, 0.8]	
	All ASD	0.78, [0.75, 0.83]	T(29, 22) = 0.72, p = 0.63
	All CON	0.77, [0.74, 0.81]	

Mean and interquartile range (IQR) for Dice coefficients are presented for each group of subjects and for each of the automated segmentation methods (compared to manual segmentation). Independent T-tests were conducted to assess whether groups exhibited a statistically significant difference in Dice coefficient. P-values are corrected for multiple comparisons using FDR-correction.

### Probabilistic ASCHOPLEX provides a metric of model generalizability

**Fig. 4:**
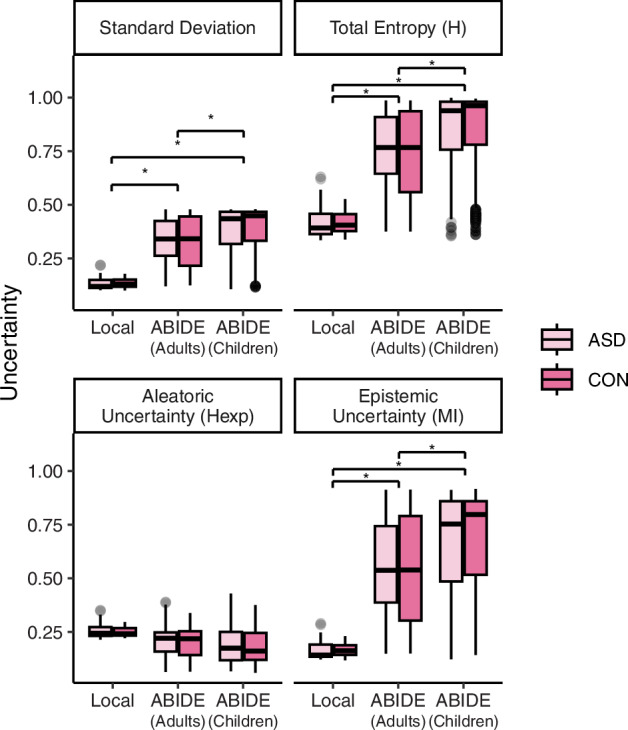
Model uncertainty computed for the different groups of subjects (adults from local dataset, adults from ABIDE and children from ABIDE). We computed four uncertainty metrics: (i) Standard deviation of the predictions, (ii) total predicted entropy (H), (iii) aleatoric uncertainty proxy (expected per sample entropy), and (iv) epistemic uncertainty (mutual information). ASD: Autism Spectrum Disorder, CON: Controls, Adults: 18 years old or older, Children: younger than 18 years old.

To assess model generalizability, four uncertainty metrics were calculated for each subject in the local and ABIDE datasets. The uncertainty metrics are displayed in Fig. 4 for the local dataset (consisting of adults) and for age subgroups (adults vs. children) for the ABIDE datasets.

We used a linear mixed effects model to test for fixed effects of diagnosis, cohort, and diagnosis x cohort interaction on each model uncertainty metric and included a random effect for scanning site [uncertainty ~ diagnosis * cohort + (1 | site)].

For standard deviation, the model detected a main effect of cohort such that standard deviation was greater for the two ABIDE cohorts compared to the local dataset (ABIDE Adults: b = 0.22, t = 3.15, p < 0.01; ABIDE Children: b = 0.27, t = 3.84, p < 0.001). The model did not detect a main effect of diagnosis or an interaction between cohort and diagnosis. Post-hoc t-tests demonstrated that while the segmentation procedure showed increased standard deviation when generalized to the ABIDE dataset (Local vs. ABIDE Adults: T(53, 529) = −3.07, p < 0.05; Local vs. ABIDE Children: T(53, 1273) = −3.84, p < 0.01), ABIDE children showed significantly higher standard deviation compared to ABIDE adults (ABIDE Adults vs. ABIDE Children: T(529, 1273) = −8.41, p < 0.001).

For total entropy, the model showed similar results. There was a main effect of cohort such that entropy was greater for the two ABIDE cohorts compared to the local dataset (ABIDE Adults: b = 0.365, t = 3.31, p < 0.01; ABIDE Children: b = 0.45, t = 4.09, p < 0.001). As was the case for standard deviation, the model did not detect a main effect of diagnosis or interaction between cohort and diagnosis on entropy. Post-hoc t-tests demonstrated that while the segmentation procedure showed increased entropy when generalized to the ABIDE dataset (Local vs. ABIDE Adults: T(53, 529) = −3.25, p < 0.01; Local vs. ABIDE Children: T(53, 1279) = −4.14, P < 0.001), ABIDE children showed significantly higher entropy compared to ABIDE adults (ABIDE Adults vs. ABIDE Children: T(529, 1279) = −9.13, p < 0.001).

We then decomposed entropy into its epistemic component and an irreducible predictive component (aleatoric uncertainty proxy), to investigate whether the increased uncertainty observed in the ABIDE cohort reflected model uncertainty or variability that cannot be reduced under the learned model. The model examining aleatoric uncertainty proxy did not show a significant main effect of cohort (ABIDE Adults: b = −0.071, t = −1.27, p = 0.18; ABIDE Children: b = −0.82, t = −1.59, p = 0.12), main effect of diagnosis (b = −0.0077, t = −0.54, p = 0.59), or interaction (b = −0.0089, t = 0.59, p = 0.55). For epistemic uncertainty, the model demonstrated a main effect of cohort such that epistemic uncertainty was greater for the ABIDE cohorts compared to the local dataset (ABIDE Adults: b = 0.44, t = 2.78, p < 0.01; ABIDE Children: b = 0.53, t = 3.39, p < 0.01). Post-hoc t-tests demonstrated that while the segmentation procedure showed higher epistemic uncertainty when generalized to the ABIDE dataset (Local vs. ABIDE Adults: T(53, 529) = −2.70, p < 0.05; Local vs. ABIDE Children: T(53, 1273) = −3.38, p < 0.01), ABIDE children showed significantly higher entropy compared to ABIDE adults (ABIDE Adults vs. ABIDE Children: T(529, 1273) = −7.90, p < 0.001).

## Discussion

In this study we demonstrate accurate automatic segmentation of the choroid plexus, which has emerged as a region of interest in ASD due to its neuroimmune functions, by using the published deep learning algorithm ASCHOPLEX [25] in two separate ASD cohorts. We show the finetuned model has high similarity with manual segmentation and performs well for both ASD and CON groups. Furthermore, we show that a probabilistic version of the algorithm can be used to assess model performance.

Using both a local dataset that included previously manually labeled “ground-truth” segmentations [21] and the ABIDE dataset [31, 32], we applied the original deterministic version of ASCHOPLEX [25] and an adapted probabilistic implementation that enables quantifying the algorithm’s uncertainty. The latter provides an additional measure to assess model performance (by quantifying model uncertainty) without “ground truth” labels. Knowledge of model uncertainty is a major advantage for real world scenarios, where a ground truth reference (such as a manual segmentation) is not available, as is the case in this study with the ABIDE dataset. Our results demonstrate that a probabilistic conversion at the finetuning stage may be sufficient to obtain uncertainty metrics from foundational models that were initially implemented as deterministic. By enabling dropout during finetuning and inference, we introduced stochasticity that allows meaningful estimation of uncertainty without requiring a complete overhaul of the architecture. Moreover, tuning an existing deterministic model in this way takes advantage of its pretrained knowledge and improves its interpretability, providing a practical and computationally efficient alternative to more complex Bayesian methods.

We demonstrated that ASCHOPLEX could be efficiently finetuned on a small subset of our local dataset (n = 12 with n = 6 participants for training and n = 6 participants for validation), consistent with prior work using ASCHOPLEX [25]. Without finetuning, ASCHOPLEX performed significantly better than FreeSurfer automated segmentation when compared with manual segmentation in our local cohort. Despite this overall improvement, ASCHOPLEX showed higher Dice coefficient for CON participants compared to ASD participants prior to finetuning. This performance bias was erased after finetuning ASCHOPLEX on our local dataset, underscoring the importance of finetuning for effective generalization to new cohorts. Overall, finetuning led to ceiling-level Dice coefficients that met or exceeded metrics of human inter-rater reliability [17, 23]. In studies that explicitly measured inter-rater reliability of manual choroid plexus segmentations, raters achieved inter-class correlation of 0.73 and Dice coefficients in the range of 0.6 – 0.8, respectively [17, 23]. Moreover, the Dice coefficients we observed after finetuning are similar to those achieved in the original generalization tests performed on ASCHOPLEX [25].

The performance of ASCHOPLEX remained stable when we implemented a probabilistic version of the algorithm, allowing us to quantify the algorithm’s uncertainty. Using the ABIDE I and II datasets, we observed that, as expected, the algorithm’s performance dropped significantly when confronted with data different from what it encountered during training and finetuning. We observed greater uncertainty overall across participants from the ABIDE cohort. While finetuning procedures are designed to improve generalization, finetuning can also result in over-learning [38], which could explain the extremely low uncertainty measures in participants from our local dataset. While uncertainty was significantly higher overall in the ABIDE dataset, we found the highest uncertainty levels in ABIDE children, which was significantly higher than the uncertainty for segmentations in ABIDE adults. When decomposing uncertainty into aleatoric and epistemic components, we found that the increased uncertainty in the ABIDE dataset was driven by epistemic uncertainty. This suggested that the increase in model uncertainty was not due to differences in overall data quality, but instead due to the model’s unfamiliarity with the new data. This likely results from the algorithm’s training and finetuning data, which consisted entirely of adult participants. Population studies have shown that while total intracranial volume increases dramatically throughout childhood, ventricular volume tends to stay relatively constant during childhood, with ventricular enlargements most commonly occurring in adulthood and older age [39]. The smaller lateral ventricles in children lead to reduced contrast between the choroid plexus and surrounding CSF and brain tissue. These features amplify partial volume effects and make the choroid plexus considerably more difficult for the algorithm to segment in children. Notably, there was no significant difference in uncertainty between CON and ASD participants from the ABIDE data. Our findings emphasize the importance of both finetuning and uncertainty quantification to ensure that deep learning models generalize effectively to new datasets, particularly in clinical applications where segmentation reliability is critical.

While our current framework already incorporates a theoretical separation, providing the epistemic component and an aleatoric proxy (irreducible uncertainty) through information theory metrics, it remains limited to MC Dropout and the ensemble method from the original architecture. This approach delivers a practical and efficient proxy for Bayesian inference (no architectural modifications, no full retraining) but offers only a partial view on the predictive posterior (the distribution of possible segmentations, given evidence accumulated by the model). Although costly from a computational standpoint, a natural extension of these methods would involve integrating mechanisms that explicitly encode aleatoric uncertainty such as multi-head architectures or deep gaussian processes. Pairing these approaches with ensemble or MC-dropout strategies could yield a richer representation of uncertainty by jointly addressing epistemic variability and inherently modeling data-driven noise. Future work could also explore complementary techniques, including latent-space distance metrics or topological uncertainty, to broaden the spectrum of captured insights and improve reliability when generalizing to new populations.

This new information, together with what we captured in this study, could be leveraged to detect model failures and guide QC (e.g. flagging specific subjects within large datasets such as ABIDE). A specific rule for relying on a model may feel arbitrary, but a decent heuristic could be to flag any new input data with uncertainty measures outside the interquartile range of a reference dataset or Kullback-Leibler (KL) divergence between new and reference uncertainty distributions surpassing a certain threshold. All these metrics would have to be considered group-wise, as group-level uncertainty is far more reliable than subject-level.

There are several intrinsic challenges to optimal finetuning procedures. Finetuning requires manual segmentations (for ground truth) which is time consuming and subjective. Additionally, it may require specific hardware (e.g. GPUs) and increase overall compute time. Moreover, there are currently limited published guidelines for manually identifying the choroid plexus based on MRI scans [40]. Rather, radiologists rely on their training and expertise, which could result in variability across training datasets. Developing a standard protocol for the manual segmentation of choroid plexus on MRIs would benefit the field. A further limitation of our approach is the small size of the finetuning dataset (n = 12). Although this sample size and dataset sharing across the ensemble’s models is consistent with the original ASCHOPLEX protocol [25], it limits the amount of data-driven variability available during finetuning and may increase the risk of convergence toward similar solutions across ensemble members. In finetuning settings with a low-data regime, the diversity induced by stochastic initialization (on prior training), MC Dropout, and independent finetuning steps cannot be theoretically guaranteed to avoid ensemble collapse.

Nevertheless, we empirically observe that this residual stochasticity is sufficient to induce functionally meaningful variability under domain shift. When evaluating the out-of-distribution ABIDE cohort, the increase in predictive uncertainty is dominated by the mutual information term of the BALD decomposition, while the expected entropy (aleatoric, irreducible uncertainty proxy) exhibits only a marginal increase. This behavior indicates that the observed uncertainty inflation is primarily driven by inter- and intra- (as per repeated MC samplings) model disagreement rather than by uniform increases in per-model uncertainty, suggesting that the ensemble retains sensitivity to epistemic uncertainty despite the limited and shared finetuning data. Additional future work could explore adjustments to the finetuning procedure such as larger and non-shared finetuning datasets or other diversity-enhancing strategies (e.g., bootstrapping, data-resampling), varying the number of iterations and halting criteria, including warmup and/or decay in the learning rate, and adjusting the batch size.

Of the many methods available for measuring uncertainty (for review, see [26]), we chose to implement a probabilistic model, by enabling dropout layers with a probability of 0.1. The choice of dropout probability is a compromise between maintaining the accuracy of the original deterministic model and allowing for sufficient variability to obtain uncertainty metrics. With a higher dropout probability, we could expect a greater standard deviation across model iterations and a higher overall uncertainty metric [34]. In the ideal case, where a model exhibits perfect performance (identical to ground truth), we would expect to see an uncertainty metric similar to the dropout rate [35]. Under MC-Dropout, activating dropout at test time approximates sampling from a posterior over weights. For in-distribution inputs distant from decision boundaries, the sampled subnetworks produce almost identical logits, and the softmax attenuates residual variability. The residual variance scales approximately as $p/(1-p)$ setting a dataset-specific ‘uncertainty floor’ close to the dropout rate p [34, 35]. Values near this floor indicate epistemically easy, stable predictions. This is indeed what we saw for the local dataset, where the probabilistic model achieved a high Dice coefficient, and showed an uncertainty metric close to the dropout rate of 0.1. This suggests that the method we chose provided adequate information for evaluating generalization. For the local dataset and a representative subset of ABIDE we were able to produce 100 samples per subject, effectively approaching convergence as per established practice on a subject-level. The same would be computationally prohibitive to do for the entire ABIDE cohort, where we settled on 25 samples per subject. Despite that, as shown in Figure S6, results remained consistent between 100 samples on the ABIDE subset and 25 samples on the whole dataset. This likely stems from the considered metric being computed group-wise, thus requiring a reduced number of samples, since many of the factors influencing uncertainty are shared across multiple subjects.

In our assessment of algorithm generalization in the context of the ABIDE dataset, we examined age and diagnosis as potential metrics where uncertainty may differ. As expected, we found the lowest uncertainty in adults (compared to children) and similar levels of uncertainty across diagnoses (ASD and CON). Other factors that may influence generalization include participant demographics and data acquisition parameters (e.g., site, scanner type, MRI sequences). Some of these factors likely contributed to the increased uncertainty we observed in the ABIDE dataset. Translation of automated choroid plexus segmentation to other cohorts will require training/finetuning on datasets that are based on a wide range of participant demographics and acquisition parameters. The ease of use of finetuning within the ASCHOPLEX user-interface facilitates its generalization to new datasets. Based on our findings, finetuning procedures should become standard practice when applying deep neural nets to neuroimaging contexts.

## Supplementary information

Supplementary material

SI ABIDE exclusions
