# Supplementary material for: A probabilistic deep learning approach for choroid plexus segmentation in autism spectrum disorder
Source: NPP Digit Psychiatry Neurosci. 2026 Jan 30;4:2. doi: 10.1038/s44277-026-00056-1 (PMC12858829; doi:10.1038/s44277-026-00056-1)
Supplement: Supplementary file 1 — Supplementary material [file 44277_2026_56_MOESM1_ESM.pdf]

Supplementary Methods:

Detailed Information About the ASCHOPLEX Model: The ASCHOPLEX model is an ensemble made up of five models trained on manually segmented choroid plexus images. The manual segmentations were derived from T1-weighted MPRAGE anatomical MRI datasets consisting of healthy adult controls (n = 17, age: 37.2 +/- 9.5 years) and adults with relapsing-remitting multiple sclerosis (Dataset 1: n = 29, age: 40.9 +/- 9.9 years; Dataset 2: n = 46, age: 36.7 +/- 10.1 years)[25]. When applying ASCHOPLEX to new datasets, the developers recommend using a finetuning procedure they have implemented to improve generalizability on unseen data[25]. During the finetuning procedure, as per the original ASCHOPLEX paper[25], all model parameters were unfrozen and hyperparameters were: (i) learning rate = 1e-4, (ii) weight decay = 1e-5, (iii) batch size = 1, (iv) max iterations = 10,000 (vs. 20,000 for original training), (v) Adam-W optimizer. No k-fold cross-validation was performed at this stage; model selection was based on peak validation Dice. To apply the model in the context of ASD, we performed finetuning on a representative subsample of our local dataset, following the original ASCHOPLEX paper recommendations: 5 subjects for training and 5 for validation restored in-distribution level performance, with our choice landing on 6 and 6 to ensure representativeness. The sample used for finetuning consisted of twelve subjects: six subjects for training (n = 3 ASD; [2 males, 1 female], n = 3 CON [2 males, 1 female]), and six subjects for validation (n = 3 ASD; [2 males, 1 female], n = 3 CON [2 males, 1 female]). After finetuning, we tested the model's performance on the remaining 53 subjects using the Dice similarity coefficient to quantify the model's accuracy. The 53 subjects in the test set consist of 30 ASD (20 males, 10 females) and 23 CON (15 males, 8 females).

Assessing Model Performance on the Local Dataset: In addition to calculating the Dice coefficient (see **Figure 3**), we calculated volume similarity, Hausdorff distance, and the Pearson correlation of volumes between automated and manual segmentation methods to assess model performance (see **Figure S5** and **Tables S1, S2, and S3**). Definitions for volume similarity and Hausdorff distance were adapted from Taha et al. (2015)[40].

Volume Similarity: For each subject and automated segmentation method, volume similarity was defined as:

$$VS = 1 - \frac{||S_a| - |S_m||}{|S_a| + |S_m|},$$

where $|S_a|$ is the number of voxels labeled as choroid plexus by the automated segementation method, and $|S_m|$ is the number of voxels labeled as choroid plexus by the manual segmentation. This was implemented using custom python code. Volume similarity statistics are presented in **Figure S5a,b and Table S1**.

Hausdorff Distance: For each subject and automated segmentation method, Hausdorff distance was defined as:

$$HD(A, M) = \max\,(h(A, M), h(M, A)$$

where h(M,A) is the directed Hausdorff distance defined by:

$$h(A, M) = max_{a \in A} min_{m \in M} ||a - m||$$

where ||a – m|| is the Euclidean distance between point a in the automated segmentation and point m in the manual segmentation. This was implemented using the directed_hausdorff function from the scipy.spatial.distance library in Python. Hausdorff distances statistics are presented in **Figure S5c,d and Table S2**.

Pearson Correlation of Volumes: For each automated segmentation method, Pearson correlation between choroid plexus volumes determined by automated and manual methods was calculated across subjects. Pearson correlation statistics are presented in **Figure S5e,f and Table S3**.

Supplementary Results:

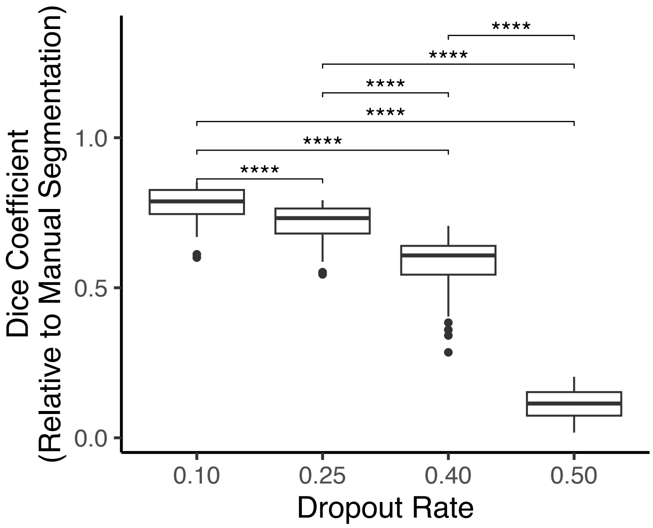

Figure S1. The probabilistic ASCHOPLEX model was empirically tested using dropout rates of 0.1, 0.25, 0.4, and 0.5. Dice Coefficients for the 53 held-out participants are plotted for each dropout rate. Each stepwise increase in dropout rate showed a statistically significant decline in segmentation performance, as measured by Dice coefficient. To minimize losses in segmentation performance while still allowing for enough variability to gather significantly detailed uncertainty metrics, we ultimately selected a dropout rate of 0.1. (* indicates $p < 0.05$ according to a paired-samples T-test).

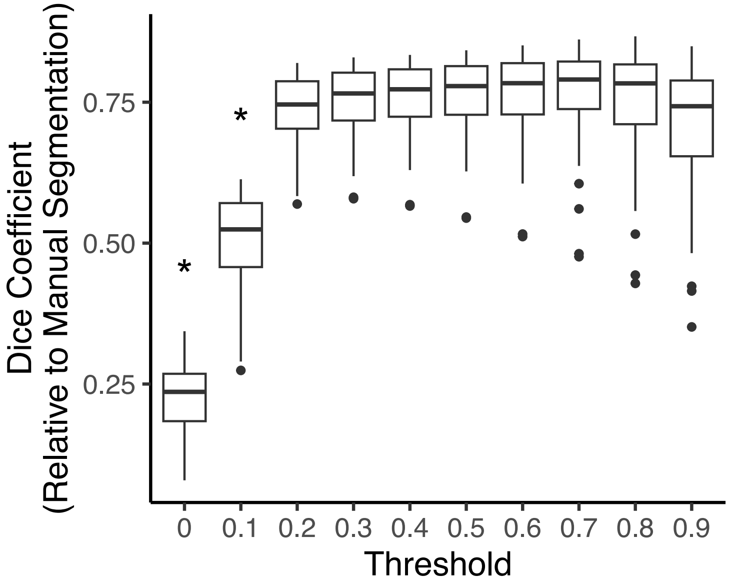

Figure S2. The probabilistic ASCHOPLEX model was empirically tested using thresholds ranging from 0 – 0.9. Dice Coefficients for the 53 held-out participants are plotted for each threshold. Thresholds of 0 and 0.1 showed significantly lower Dice coefficients compared to each of the other thresholds. Thresholds in the range of 0.2 – 0.9 showed no statistically significant difference from each other. The threshold of 0.5, which was chosen for the final segmentation procedure, falls within the range of thresholds that show the best Dice coefficients and adheres to standard procedures of classification based on majority voting procedures. (* indicates a threshold level that showed a significantly different Dice coefficient compared to all other thresholds, according to paired-samples T-tests.)

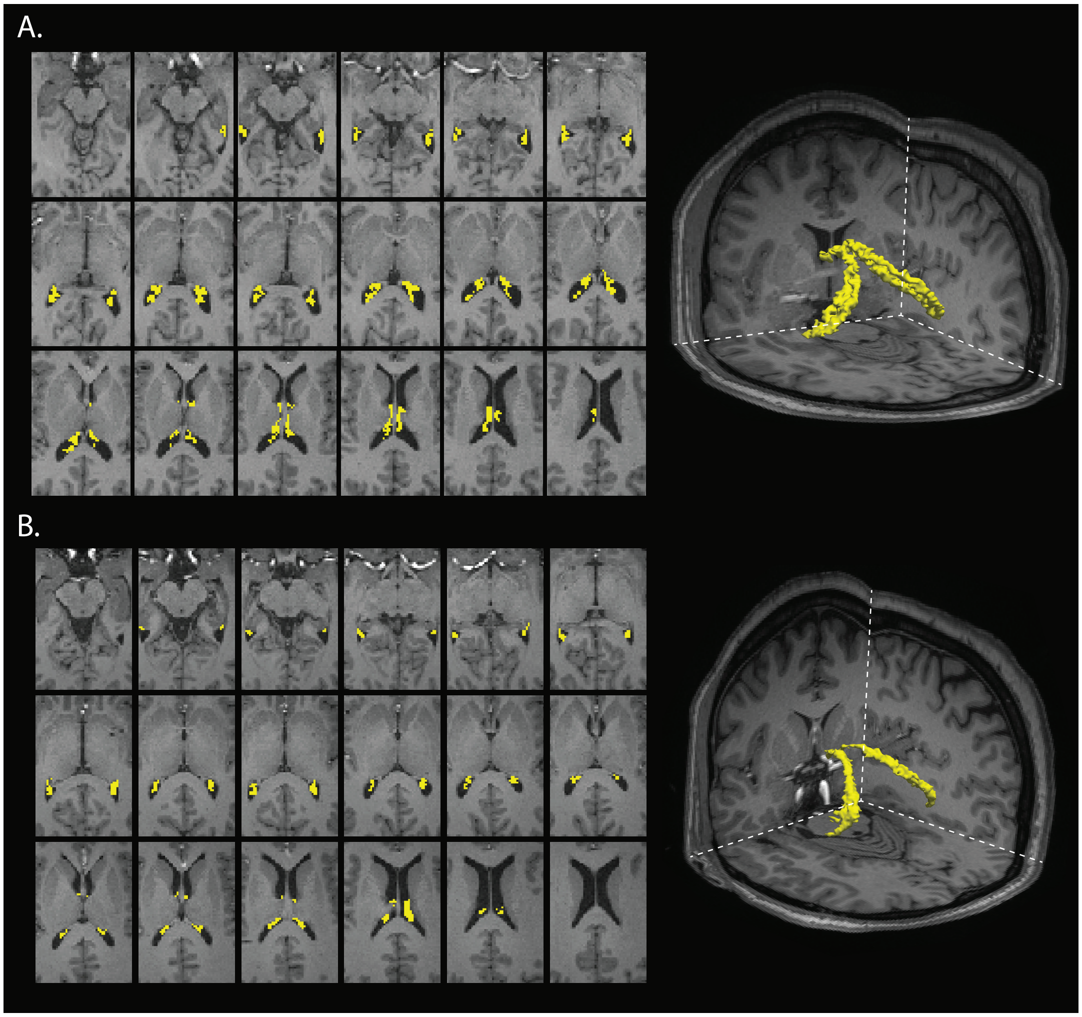

Figure S3. Examples of manual segmentations across axial slices (left) and as 3D surfaces (right) in a representative ASD subject (**A**) and a representative CON subject (**B**). Yellow = choroid plexus.

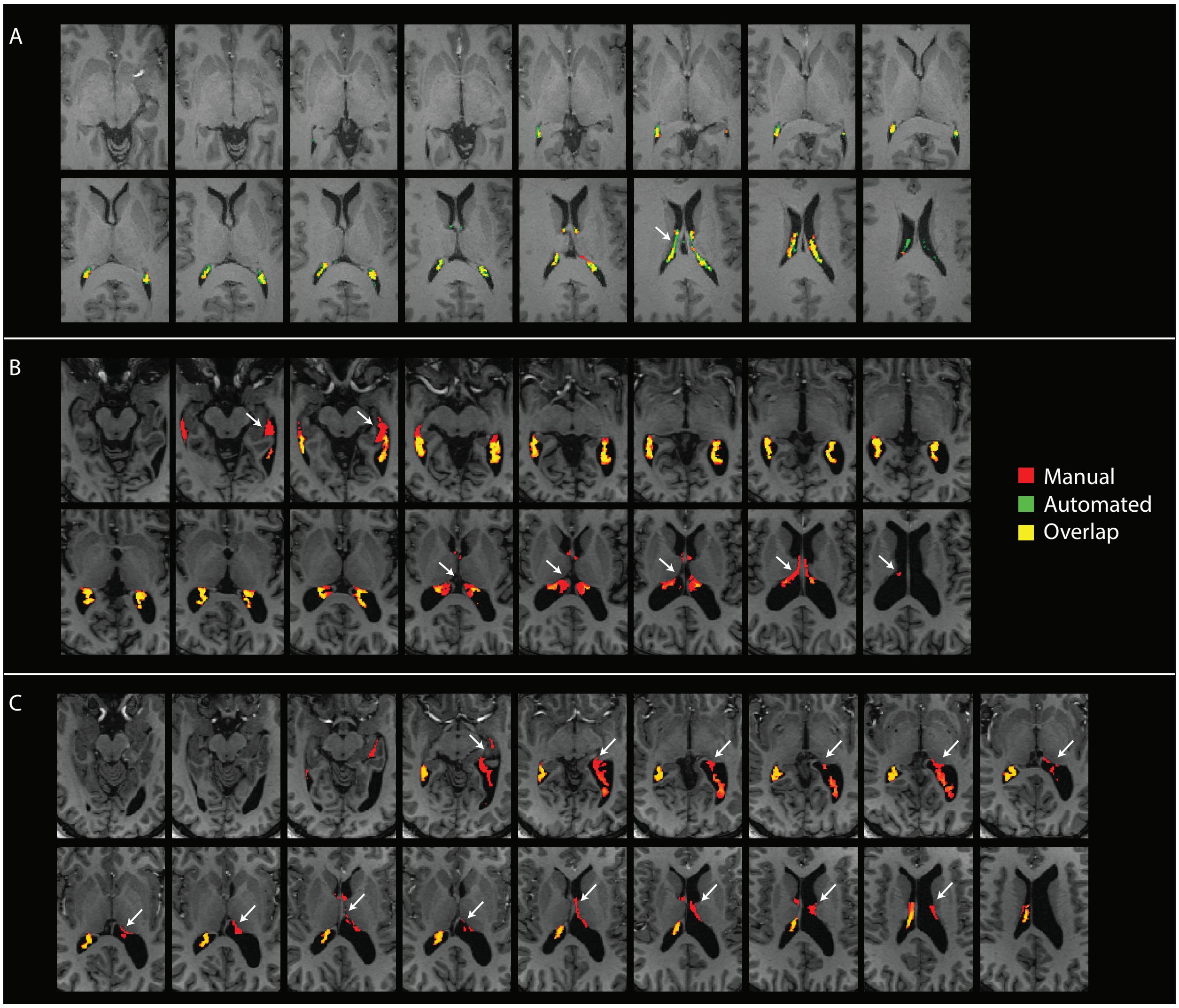

Figure S4. Failure Characterization. Representative images (axial slices) of common failures in the algorithm's segmentation of choroid plexus. **(A)** An adult CON subject where the algorithm "filled in" non-contiguous voxels with a choroid plexus label to connect two regions that were labeled choroid plexus in the manual segmentation. **(B)** An adult ASD subject where the algorithm missed choroid plexus voxels in the most superior and inferior slices. **(C)** An adult ASD subject with asymmetrical lateral ventricles, where the algorithm failed to label the choroid plexus in the enlarged ventricle. Red = voxels labeled as choroid plexus via manual segmentation. Green = voxels labeled choroid plexus via automated segmentation. Yellow = overlap.

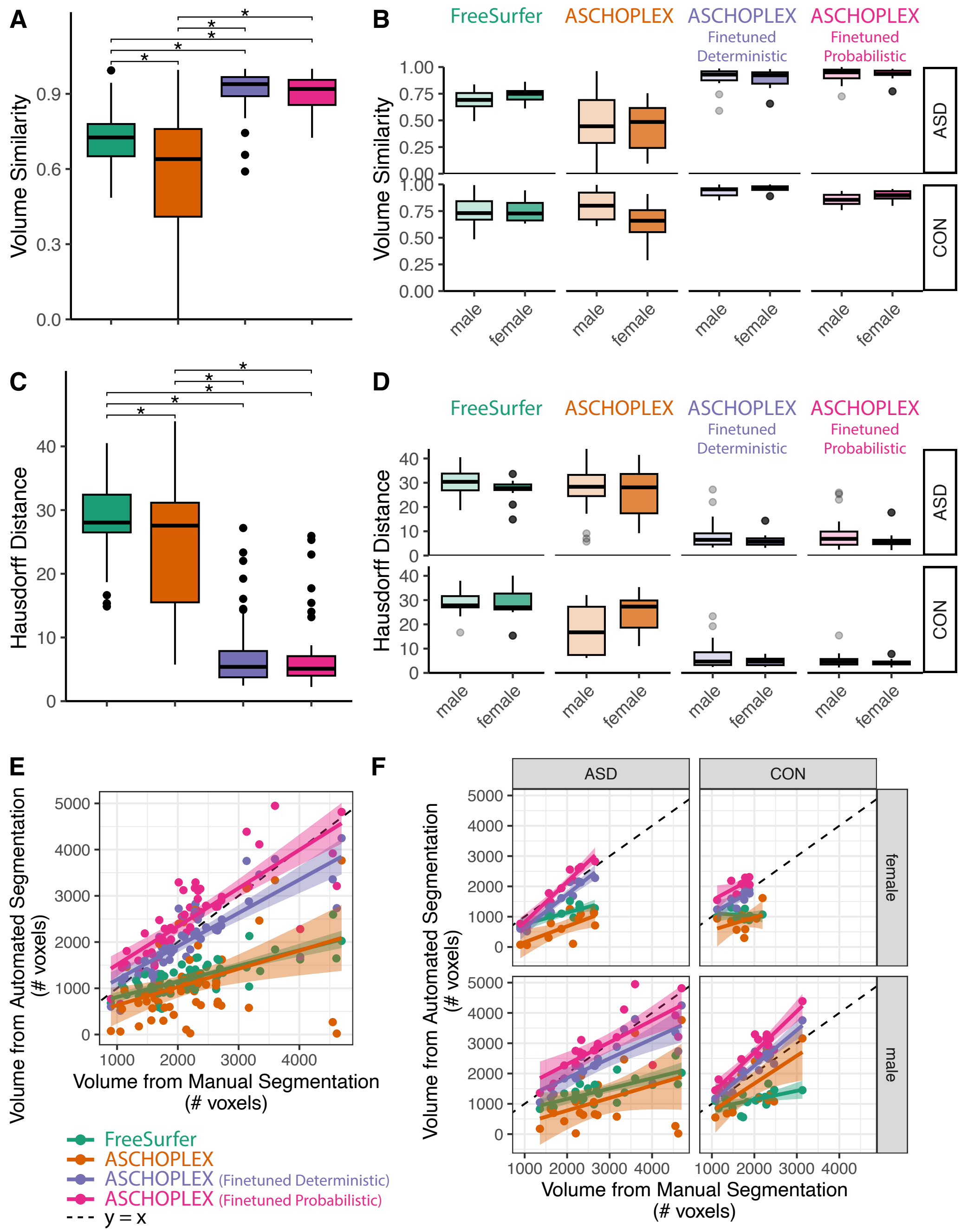

Figure S5. Volume similarity (A) and Hausdorff distance (C) metrics for the automated segmentation methods vs. manual segmentation in the 53 held-out participants from the local dataset. Volume similarity (B) and Hausdorff distance (D) metrics compared across sex and diagnosis for each of the segmentation methods for participants from the local dataset. Scatter plots showing Pearson correlation between the choroid plexus volume calculated from manual segmentation and each of the automated segmentation methods (E). In the final panel, the volume comparison is separated out by sex and diagnosis (F). Green = FreeSurfer, Orange = ASCHOPLEX (no finetuning), Purple = ASCHOPLEX (Finetuned & Deterministic), Pink = ASCHOPLEX (Finetuned & Probabilistic).

Table S1. Model Performance Bias Assessment (Volume Similarity)

Model	Group	Mean [IQR]	T-Test
FreeSurfer	ASD Female	0.74, [0.7, 0.78]	T(11, 23) = 1.75, p = 0.25
	ASD Male	0.69, [0.63, 0.76]	
	CON Female	0.76, [0.66, 0.83]	T(9, 18) = 0.43, p = 0.77
	CON Male	0.74, [0.67, 0.84]	
	All ASD	0.7, [0.65, 0.76]	T(35, 28) = -1.53, p = 0.13
	All CON	0.75, [0.66, 0.83]	
ASCHOPLEX	ASD Female	0.44, [0.24, 0.62]	T(9, 19) = -0.65, p = 0.77
	ASD Male	0.5, [0.33, 0.71]	
	CON Female	0.65, [0.55, 0.76]	T(7, 14) = -2.14, p = 0.25
	CON Male	0.8, [0.67, 0.92]	
	All ASD	0.48, [0.31, 0.65]	T(29, 22) = -4.26, p < 0.001
	All CON	0.74, [0.65, 0.91]	
ASCHOPLEX Finetuned, Deterministic	ASD Female	0.89, [0.84, 0.95]	T(9, 19) = -0.51, p = 0.77
	ASD Male	0.91, [0.88, 0.96]	
	CON Female	0.96, [0.95, 0.98]	T(7, 14) = 1.61, p = 0.25
	CON Male	0.93, [0.9, 0.96]	
	All ASD	0.9, [0.87, 0.96]	T(29, 22) = -2.16, p = 0.05
	All CON	0.94, [0.9, 0.97]	
ASCHOPLEX Finetuned, Probabilistic	ASD Female	0.93, [0.93, 0.97]	T(9, 19) = 0.07, p = 0.94
	ASD Male	0.93, [0.89, 0.98]	
	CON Female	0.89, [0.87, 0.94]	T(7, 14) = 1.64, p = 0.25
	CON Male	0.85, [0.82, 0.9]	
	All ASD	0.93, [0.9, 0.97]	T(29, 22) = 3.46, p < 0.001
	All CON	0.87, [0.83, 0.91]	

Mean and interquartile range (IQR) for volume similarity are presented for each group of subjects for each of the automated segmentation methods (compared to manual segmentation). Independent T-tests were conducted to assess whether groups exhibited a statistically significant difference in volume similarity. P-values are corrected for multiple comparisons using FDR-correction.

Table S2. Model Performance Bias Assessment (Hausdorff Distance)

Model	Group	Mean [IQR]	T-Test
FreeSurfer	ASD Female	27.03, [26.99, 29.3]	T(11, 23) = -1.85, p = 0.31
	ASD Male	30.24, [26.87, 33.77]	
	CON Female	28.86, [26.14, 32.7]	T(9, 18) = 0.08, p = 0.94
	CON Male	28.67, [27, 31.64]	
	All ASD	29.17, [26.87, 32.63]	T(35, 28) = 0.33, p = 0.74
	All CON	28.73, [26.5, 31.83]	
ASCHOPLEX	ASD Female	26.34, [17.4, 33.61]	T(9, 19) = -0.23, p = 0.94
	ASD Male	27.26, [24.5, 33.24]	
	CON Female	24.98, [18.65, 29.85]	T(7, 14) = 2.17, p = 0.31
	CON Male	16.95, [7.38, 27.26]	
	All ASD	26.93, [19.76, 34.06]	T(29, 22) = 2.38, p < 0.05
	All CON	20.29, [10.64, 28.35]	
ASCHOPLEX Finetuned, Deterministic	ASD Female	6.4, [4.46, 7.09]	T(9, 19) = -1.27, p = 0.34
	ASD Male	8.65, [4.53, 9.14]	
	CON Female	4.71, [3.19, 5.85]	T(7, 14) = -1.56, p = 0.34
	CON Male	7.5, [3.24, 8.5]	

	All ASD	7.9, [4.41, 8.18]	T(29, 22) = 0.9, p = 0.5
	All CON	6.53, [3.24, 7.1]	
ASCHOPLEX Finetuned, Probabilistic	ASD Female	6.47, [4.69, 6.5]	T(9, 19) = -1.3, p = 0.34
	ASD Male	9.23, [4.44, 9.88]	
	CON Female	4.42, [3.49, 4.71]	T(7, 14) = -0.88, p = 0.52
	CON Male	5.32, [3.46, 5.71]	
	All ASD	8.31, [4.5, 8.15]	T(29, 22) = 2.49, p < 0.05
	All CON	5.01, [3.46, 5.42]	

Mean and interquartile range (IQR) for Hausdorff distance are presented for each group of subjects for each of the automated segmentation methods (compared to manual segmentation). Independent T-tests were conducted to assess whether groups exhibited a statistically significant difference in Hausdorff distance. P-values are corrected for multiple comparisons using FDR-correction.

Table S3. Pearson Correlation of Manually vs. Automatically Segmented Volumes

Sample	FreeSurfer	ASCHOPLEX	ASCHOPLEX (Finetuned, Deterministic)	ASCHOPLEX (Finetuned, Probabilistic)
All	0.76, [0.63, 0.85]	0.37, [0.12, 0.58]	0.81, [0.69, 0.89]	0.81, [0.69, 0.89]
ASD male	0.75, [0.49, 0.88]	0.39, [-0.08, 0.71]	0.77, [0.49, 0.9]	0.76, [0.47, 0.9]
CON male	0.54, [0.12, 0.8]	0.62, [0.13, 0.86]	0.93, [0.8, 0.98]	0.93, [0.81, 0.98]
ASD female	0.8, [0.42, 0.94]	0.69, [0.16, 0.91]	0.96, [0.85, 0.99]	0.97, [0.88, 0.99]
CON female	-0.17, [-0.72, 0.51]	0.37, [-0.34, 0.81]	0.79, [0.2, 0.96]	0.73, [0.06, 0.95]

Pearson correlations of volumes calculated by the automated and manual segmentation procedures are displayed. Ranges in brackets indicate 95% confidence intervals.

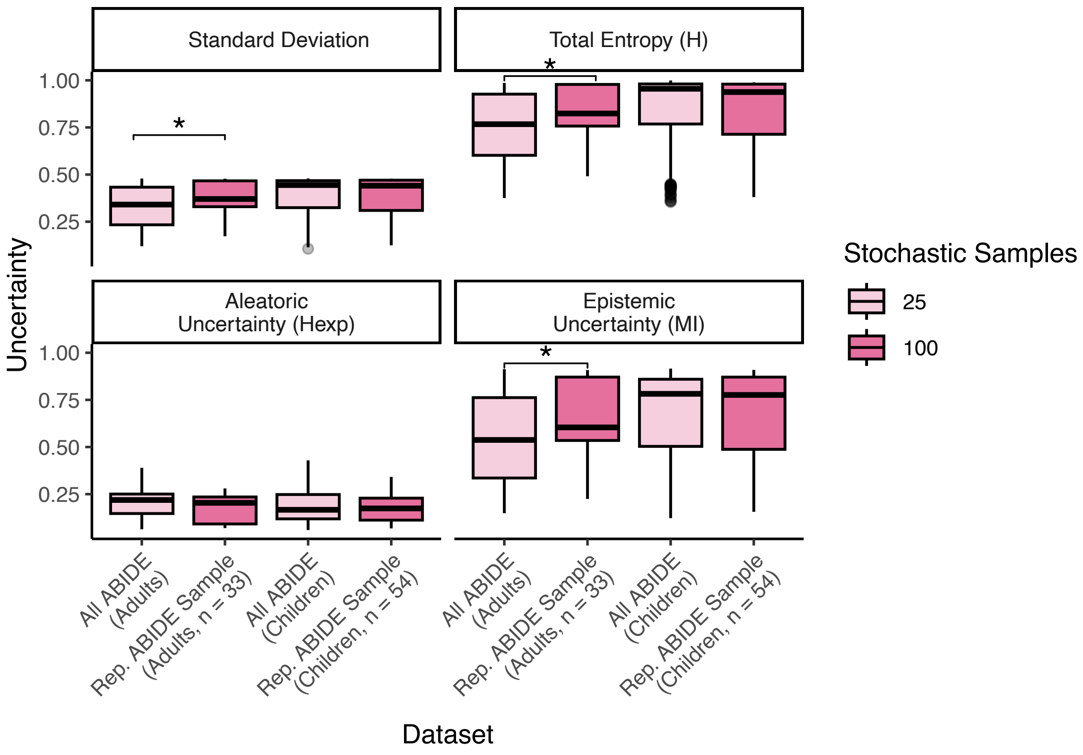

Fig. S6. For a representative subset of the ABIDE dataset (86 subjects; n = 33 adults and n = 54 children) we generated 100 total stochastic samples per subject (20 Monte-Carlo runs x 5 ensemble models). For the full ABIDE dataset (n = 1,802), we used a lighter scheme of 25 samples per subject (5 Monte-Carlo runs x 5 models). Although significant differences in uncertainty measures are visible in adults across stochastic sampling rates, the qualitative pattern of results remains consistent, whereby across sampling schemes, children show a qualitatively higher uncertainty compared to adults.

Table S4.

Data Dictionary		
**column name**	**format**	**description**
subject	"site_" numbers	unique subject identifier
age	numerical	Subject age in years
site	string	unique site identifier
dataset	ABIDEI or ABIDEII	indicates if subject was included in ABIDE I or ABIDE II data release
dx_gorup	ASD or CON	ASD: subject diagnosed with autism spectrum disorder; CON: subject not diagnosed with autism spectrum disorder
excluded	1 or 0	1: Poor T1w structural scan quality, exclude this participant from analyses; 0: Acceptable T1w structural scan quality
Note: For subjects with longitudinal data, only the first scan was checked for quality control.		

Table S4. Data dictionary describing the format of each column in the supplementary data file SI_ABIDE_exclusions.xlsx
